# Cystoid maculopathy is a frequent feature of Cohen syndrome-associated retinopathy

**DOI:** 10.1038/s41598-021-95743-8

**Published:** 2021-08-12

**Authors:** Pierre-Henry Gabrielle, Laurence Faivre, Isabelle Audo, Xavier Zanlonghi, Hélène Dollfus, Alberta A. H. J. Thiadens, Christina Zeitz, Grazia M. S. Mancini, Yaumara Perdomo, Saddek Mohand-Saïd, Eléonore Lizé, Vincent Lhussiez, Emeline F. Nandrot, Niyazi Acar, Catherine Creuzot-Garcher, José-Alain Sahel, Muhammad Ansar, Christel Thauvin-Robinet, Laurence Duplomb, Romain Da Costa

**Affiliations:** 1 Department of Ophthalmology, University Hospital, 14 rue Paul Gaffarel, 21079 Dijon, France; 2 Inserm, UMR1231, Equipe GAD, Université de Bourgogne Franche Comté, Bâtiment B3, 15 Boulevard du Maréchal de Lattre de Tassigny, 21079 Dijon Cedex, France; 3 FHU TRANSLAD, CHU Dijon, 21000 Dijon, France; 4 Centre de Référence Anomalies du Développement et Syndromes Malformatifs, CHU Dijon, 21000 Dijon, France; 5 Sorbonne Université, INSERM, CNRS, Institut de La Vision, 17 rue Moreau, 75012 Paris, France; 6 CHNO Des Quinze-Vingts, DHU Sight Restore, INSERM-DGOS CIC 1423, 75012 Paris, France; 7 Maladies Rares, Service d’Ophtalmologie, CHU Rennes, 2 rue Henri Le Guilloux, 35033 Rennes, France; 8 Centre de Référence Pour Les Affections Rares en Génétique Ophtalmologique (CARGO), FSMR SENSGENE, ERN-EYE, Hôpitaux Universitaires de Strasbourg, 67000 Strasbourg, France; 9 Laboratoire de Génétique Médicale, Inserm, UMR1112, Institut de Génétique Médicale D’Alsace, Université de Strasbourg, 67000 Strasbourg, France; 10 Department of Ophthalmology, Erasmus MC, 3015 Rotterdam, The Netherlands; 11 Department of Clinical Genetics, Erasmus MC, 3015 Rotterdam, The Netherlands; 12 Centre Des Sciences du Goût Et de L’Alimentation, AgroSup Dijon, CNRS, INRA, Université Bourgogne Franche-Comté, 9E Boulevard Jeanne d’Arc, 21000 Dijon, France; 13 Department of Ophthalmology, The University of Pittsburgh School of Medicine, Pittsburgh, PA 15213 USA; 14 Institute of Molecular and Clinical Ophthalmology Basel, 4031 Basel, Switzerland; 15 Centre de Référence Déficiences Intellectuelles de Causes Rares, CHU Dijon, 21000 Dijon, France; 16 Department of Ophthalmology, Jules-Gonin Eye Hospital, University of Lausanne, 1004 Lausanne, Switzerland

**Keywords:** Retinal diseases, Hereditary eye disease, Retina, Medical genetics

## Abstract

Cohen syndrome (CS) is a rare syndromic form of rod-cone dystrophy. Recent case reports have suggested that cystoid maculopathy (CM) could affect CS patients with an early onset and high prevalence. Our study aims at improving our understanding and management of CM in CS patients through a retrospective case series of ten CS patients with identified pathogenic variants in *VPS13B*. Longitudinal optical coherence tomography (OCT) imaging was performed and treatment with carbonic anhydrase inhibitors (CAI) was provided to reduce the volume of cystoid spaces. CM affected eight out of ten patients in our cohort. The youngest patient showed a strong progression of macular cysts from the age of 4.5 to 5 years despite oral CAI medication. Other teenage and young adult patients showed stable macular cysts with and without treatment. One patient showed a moderate decrease of cystoid spaces in the absence of treatment at 22 years of age. Through a correlative analysis we found that the volume of cystoid spaces was positively correlated to the thickness of peripheral and macular photoreceptor-related layers. This study suggests that CAI treatments may not suffice to improve CM in CS patients, and that CM may resolve spontaneously during adulthood as photoreceptor dystrophy progresses.

## Introduction

Cohen syndrome (CS, MIM #216550) is a rare autosomal recessive disorder caused by single-nucleotide variants and chromosomic rearrangements affecting the *VPS13B* (*Vacuolar Protein Sorting 13 Homolog B*) gene (MIM *607817)^1–4^. The gene ubiquitously encodes for the VPS13B protein implicated in endolysosomal transport^5,6^ and protein glycosylation^7^. Hallmarks of the disorder^8,9^ include a characteristic facial appearance, childhood hypotonia, acquired microcephaly, intellectual disability, neutropenia^10,11^, predispositions to type II diabetes^12^ and various ophthalmic alterations^13^. After its identification in 1973^14^, advances in the characterization of the ophthalmic features were initially hindered by the low numbers of patients identified, the lack of genetic diagnosis and the absence of imaging technique of the retinal architecture. Later, substantial variability in the ophthalmic manifestations was reported^15–20^. Early-onset pathological myopia and retinal dystrophy are the most consistent findings associated with the disease. The penetrance of cataract is also important in adult CS patients^21^. Interestingly, cataract was also reported in several CS children^20^. Other ophthalmic features have a lower penetrance and seem to depend on additional factors such as the genetic background^20,21^. Those features include microphthalmia, astigmatism, strabismus, nystagmus, corneal ectasia, iris atrophy, lens subluxation, raised intraocular pressure and glaucoma^17–19,22^.

CS-associated retinopathy presents with typical rod-cone dystrophy features, also known as retinitis pigmentosa (RP), including night blindness, loss of peripheral vision and progressive retinal degeneration. Fundus abnormalities often progress quickly to display retinal vessel attenuation, optic disc pallor, bull’s eye macular atrophy, and peripheral pigmentary changes^13,17^. Full-field electroretinogram (ERG) is often markedly attenuated before the age of 5 years and undetectable afterwards^18^. Recently, nine case reports using spectral-domain optical coherence tomography (OCT) provided initial information on the macular changes affecting CS patients^23–31^. Out of the 17 patients examined so far, 15 presented with schisis-like changes and cystoid spaces in the macula. Interestingly, two of these patients underwent fluorescein angiography and showed these macular cysts to be non-leaking^24,27^. While cystoid changes in CS patients have sometimes been referred to as Cystoid Macular Edemas (CME) in previous reports, they will be referred to as cystoid maculopathy (CM) in this study due to the absence of clear etiology. In other RP forms, the detection rate of CM is about 10–20% through fluorescein angiography and as high as 50% in cohorts examined with OCT imaging^32,33^. In CS patients, CM mostly affected the fovea but sometimes expanded to more peripheral macular regions. CM may cause blurred vision and therefore contribute to the apparent difficulty of CS patients to interact with their environment. While there is not always a correlation between the macular structure and the visual acuity (VA), the development of a CM in association with VA decrease would indicate that treating CM could be beneficial to the patient vision. However, the association of CM development and VA decrease has never been established in CS since almost all patients examined through OCT imaging already had extensive CM at first examination. This is likely due to an early onset of CM in infants with CS^23,24,27,30^. In the absence of data, it remains possible that treating CM in CS patients may contribute to improving their VA.

Multiple mechanisms may be responsible for the development of CM^34^, including vitreous traction, disruption of the blood-retinal barrier, inflammatory events in the retina, or dysfunctional water/ion transport across the plasma membrane of Müller glia and retinal pigmented epithelial (RPE) cells. Several pharmacological treatments have been developed accordingly. The gold standard treatment often consists of topical and oral carbonic anhydrase IV inhibitors (CAIs), assumed to increase water transport across the RPE and Müller cells^35–37^. Other therapeutic approaches based on glucocorticoids, non-steroid anti-inflammatory drugs, anti-VEGF as well as vitreoretinal surgery have been sporadically used in interventional case series^33,38^. While some approaches provided encouraging results (e.g. vitreoretinal surgery, Ozurdex^®^), the use of others is controversial due to their mode of administration and action, link with elevated intraocular pressure or cataract development, and poor efficacy^33,39^.

Despite the relative high rate of CM in CS-associated retinopathy, there is still a lack of description and long-term longitudinal follow-up data to improve our understanding and management of this macular complication. Using OCT imaging on a cohort of 10 CS patients, we aimed at defining the frequency of CM secondary to CS-associated retinopathy and its evolution through time with or without treatment. We also explored the previously hypothesized genotype–phenotype correlation of CS ophthalmic issues to gain wider insights into CM's pathophysiological course.

## Methods

### Patients and ophthalmic examination

This multicentric study is based on a retrospective analysis that includes 1 Dutch and 9 French CS patients with previously identified disease-causing *VPS13B* variants^7,12^. Their clinical and molecular data are summarized in Supplementary Table S1. Written informed consent was obtained from each participant. For patients under 18 years old or adult with intellectual disability, informed consent was obtained from a parent and/or legal guardian. This study adhered to the tenets of the Declaration of Helsinki and has been approved by the ethics committee of Dijon University Hospital.

The diagnosis of CS-associated retinopathy was established using comprehensive ophthalmological examination and multimodal imaging, including fundus color photography (Optos, NIKON, Tokyo, Japan), autofluorescence imaging (Optos, NIKON; Spectralis, Heidelberg Engineering, Heidelberg, Germany) and OCT macular scan (Spectralis, Heidelberg Engineering; Cirrus HD-OCT, Carl Zeiss Meditec, Dublin, CA, USA). Depending on the patient’s cooperation, the ophthalmic investigation was completed with a best-corrected visual acuity test, a Goldmann perimetry and a slit-lamp examination.

### OCT analysis

**Figure 1:**
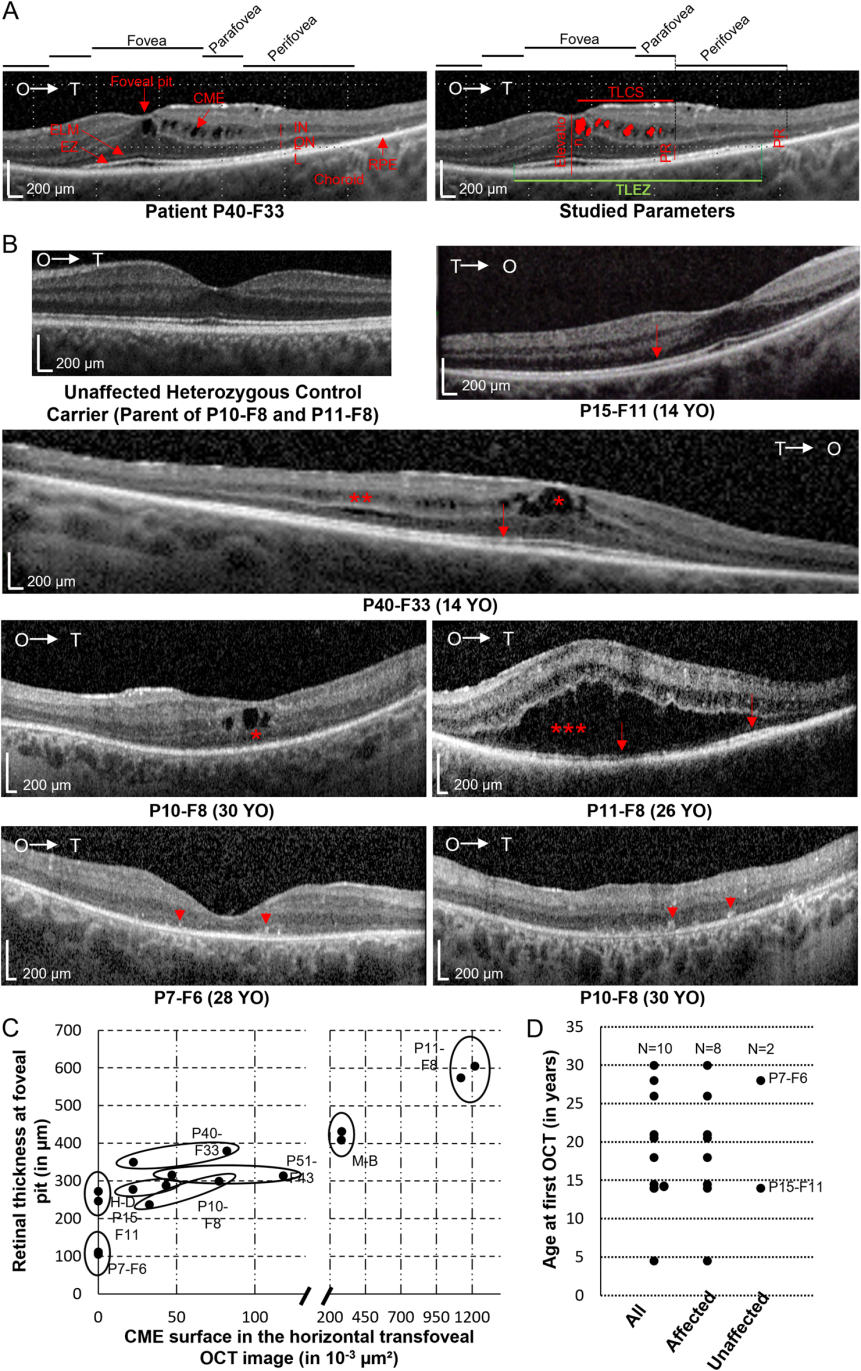
Macular lesions defined by OCT examinations in CS patients. (**A**) Parameters analyzed in this study are presented on an example of an OCT image from a CS patient (P40-F33). *CM* Cystoid Macular Edema, *ELM* External Limiting Membrane, *EZ* Ellipsoid Zone, *INL* Inner Nuclear Layer, *ONL* Outer Nuclear Layer, *PR* Photoreceptor-related layers, *TLCS* Transversal Length of Cystoid Space, *TLEZ* Transversal Length of Ellipsoid Zone. (**B**) Transfoveal OCT scans from CS patients P compared to an unaffected parent of P10-F8 and P11-F8 (upper left image) showing the variability of lesions affecting the macula. The lower right image is a parafoveal image showing hyperreflective foci in the outer retina. Arrows: Ellipsoid zone; Arrowheads: Hyperreflective foci; *CM in the INL; **CM in the ONL; ***severe discontinuation of the ONL due to CM. (**C**) Dot plot describing the variability and severity of cystoid spaces in CS patients. (**D**) Plot showing the age distribution of CS patients of this retrospective case series and the presence of CM in relation to the patients’ age.

Parameters that were analyzed from the OCT images obtained in this study are described in Fig. 1A and include: (1) presence and volume of CM; (2) retinal thickness at fovea; (3) transversal length of cystoid spaces (TLCS); (4) transversal length of preserved ellipsoid zone (TLEZ); (5) photoreceptor thickness at parafovea and perifovea. Parafoveal and perifoveal measurements were performed 1 mm and 3 mm away from the foveal pit in the temporal direction, respectively. These locations were chosen since they were in most cases not affected with cystoid spaces and allowed for an accurate measurement and correlation of the photoreceptor layer thickness with other OCT parameters. All measurements were performed using the ImageJ software by a single evaluator to avoid introducing variability between the different images. OCT images were recorded with a two-dimensional asymmetrical scale where both x and y axes measured 200 µm. To determine the volume of CM *Vcm*, we first calculated the volume *Vp* in µm^2^ of an image pixel using the equation:

1.1 $$Vp=\left(\frac{Lx}{Nx}\right)\times \left(\frac{Ly}{Ny}\right)$$

where *Lx* and *Ly* are the length in µm of the x and y axes, respectively; *Nx* and *Ny* are the numbers of pixels counted by ImageJ within the x and y axes, respectively. *Vcm* was then determined by the equation:

1.2 $$Vcm=Vp\times Np$$

where *Np* is the total number of pixels counted by ImageJ in manually defined cystoid areas.

Retinal thickness *Lr* was calculated in µm from the measurement *AB* of the retinal thickness in pixels by ImageJ. Calculations were done considering the orientation of the retina using a projection of the retinal borders on the x and y axes. The *Lr* distance corresponds to the hypotenuse AB of the right triangle ABC formed by these projections and can be calculated through a combination of Pythagoras’ and Thales’ theorems:

2 $$\begin{aligned}Lr & =\sqrt{{\left(BC\times Px\right)}^{2}+{\left(AC\times Py\right)}^{2}} \\ & =\sqrt{{\left(BC\times \left(\frac{Lx}{Nx}\right)\right)}^{2}+{\left(AC\times \left(\frac{Ly}{Ny}\right)\right)}^{2}} \\ & =\sqrt{{\left(AB\times \mathrm{sin}\left(\angle BAC\right)\times \left(\frac{Lx}{Nx}\right)\right)}^{2}+{\left(AB\times \mathrm{cos}\left(\angle BAC\right)\times \left(\frac{Ly}{Ny}\right)\right)}^{2}}\end{aligned}$$

where AB, BC and AC are distances of the sides of the right triangle ABC given in pixel length by ImageJ; *Px* and *Py* are the distances in µm of a pixel on the x and y axes, respectively.

Statistical analyses were performed using GraphPad Prism 9.0.1 (GraphPad Software, San Diego, California USA). Normality tests for the distribution of the OCT parameters were performed using D’Agostino and Pearson’s test and correlations were assessed using multivariate Spearman’s correlation analysis.

### Treatments with CAIs

Treatment decisions were at the discretion of the physician in consultation with the patient, thereby reflecting clinical practice. Patients H–D and O–Z were treated with oral CAI administration of acetazolamide 125 mg twice daily (Diamox^®^, Teofarma, Pavia, Italy). In addition to oral acetazolamide, patient M-B received trice daily a topical CAI instillation of brinzolamide 10 mg/ml (Azopt^®^, Novartis, Basel, Switzerland). The treatment response was evaluated at 6 months of treatment using OCT imaging.

## Results

### CM is a frequent but not systematic finding in CS patients

At first OCT examination, 8 out of 10 patients showed bilateral macular cystic changes consistent with the diagnosis of CM (Supplementary Table S1). Cystoid spaces mainly affected the inner nuclear layer (INL) and to lesser extent the outer nuclear layer (ONL) as well (Fig. 1A,B). Patient P11-F8 presented with a more severe retinal elevation and extension of CM within the ONL over the macular surface of both eyes at the age of 26 years (Fig. 1B). At a similar age, a single patient (P7-F6) had no CM but presented with a severely reduced retinal thickness at the foveola due to the loss of photoreceptors. The only patient who presented with no detectable macular changes was P15-F11 who was examined at the age of 14 years. To describe the range of macular lesions, we plotted for each patient the retinal thickness at the foveal pit against the surface area covered by the CM in the horizontal transfoveal images (Fig. 1C). Another parameter showing great variability between patients is the transversal length of the cystoid space. One patient showed foveal cystic lesions only, whereas two patients had lesions extending to the parafoveal region and another three patients up to the perifoveal region. In addition, the presence of CM was not solely dependent on the patients’ age (Fig. 1D). Of note, the youngest patient of our cohort (H–D) already presented with CM on his first OCT examination at the age of 4.5 years.

OCT examinations also showed typical features of Retinitis Pigmentosa (RP), including the peripheral loss of photoreceptor-related outer retinal layers as well as the presence of hyper-reflective subretinal and intraretinal dots (Fig. 1B, lower panels). While peripheral photoreceptors appeared to be affected in all patients, the extent of the involvement was again quite variable between patients. Patients P11-F8, P15-F11 and M-B presented with a preserved ellipsoid zone (EZ) almost over the entire macula whereas patients P7-F6, P10-F8 and P51-F43, had no detectable EZ even within the fovea.

### Topic and systemic administration of CAIs did not reduce or prevent the progression of CM in CS patients

**Figure 2:**
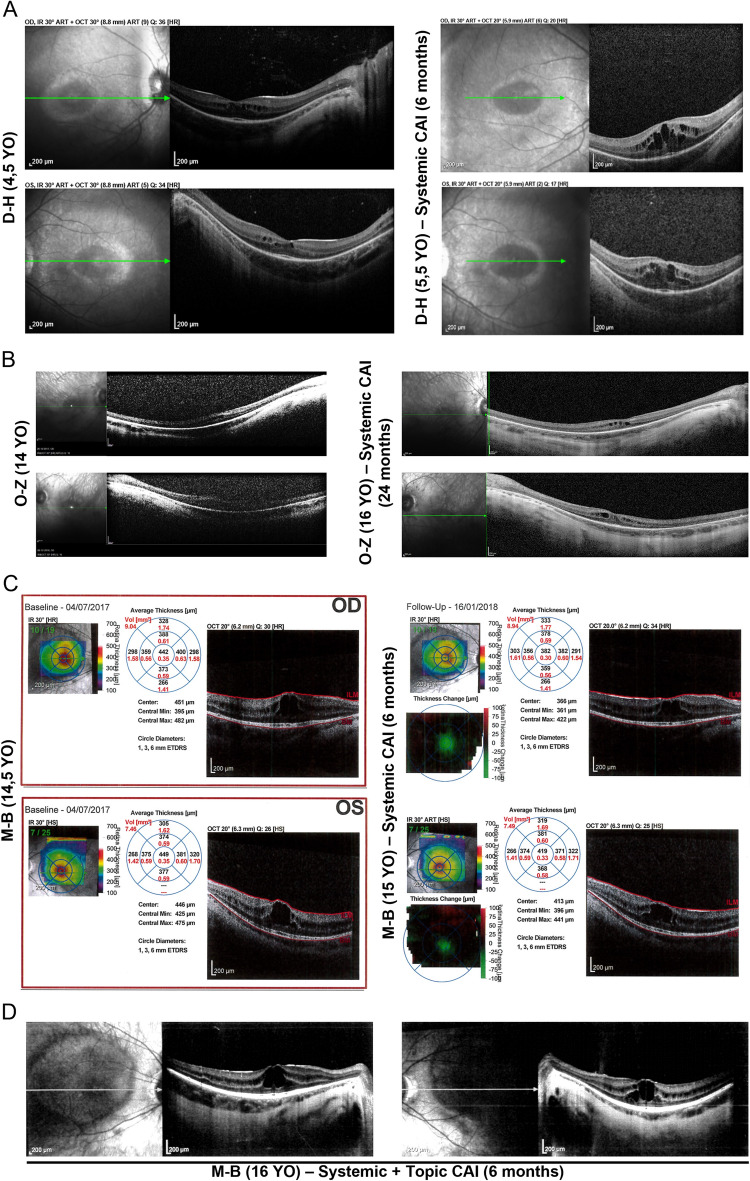
Evolution of CM upon treatment with CAIs monitored by OCT. (**A**) Transfoveal images of patient H–D before (left panels) and after (right panels) 6 months of treatment with acetazolamide. Systemic CA inhibition with acetazolamide did not prevent progression of CM in patient H–D. During the time of treatment, CM volume in transfoveal images progressed by 6-folds on the right eye and 7-folds on the left eye, and the foveal thickness was increased from 289 µm to 503 µm in the right retina and from 278 µm to 456 µm in the left retina. (**B**) Use of acetazolamide in patient O-Z was associated to a stable CM over a 24-month period. (**C**) Transfoveal images of patient M-B before (left panels) and after (right panels) 6 months of treatment with acetazolamide. OCT scans showed a 19% and 24% decrease of cystoid spaces at the fovea of the right and left retina, respectively. A 21% and 9% decrease of the foveal elevation was also measured in the right and left retina, respectively. (**D**) In an attempt to further decrease the volume of CM, acetazolamide was prescribed in combination with topical applications of brinzolamide for an additional 6 months. Follow-up OCT examination showed a return to baseline measures in terms of CM volume as well as foveal elevation.

Considering the severity of rod-cone dystrophy and the visual field constriction of CS patients, attempts were made to preserve or improve their visual acuity by reducing CM in the macula. Upon diagnosis of mild CM in patient H–D at the age of 4.5 years, a 6-month treatment with acetazolamide was prescribed. After the treatment period, the follow-up OCT examination showed a marked progression of the CM surface area in the transfoveal horizontal OCT images of both eyes (Fig. 2A). CM surface area increased by 3-folds in the right eye and 4-folds in the left eye (Supplementary Table S2). The treatment was stopped as it failed to prevent CM progression. Visual acuity could not be determined in this patient.

Acetazolamide was also prescribed to patient O-Z after the initial diagnosis at the age of 14 years. After a 6-month trial, CM volume remained stable. As of today, patient O-Z is still being treated with acetazolamide and the last OCT examination after two years of treatment showed a relatively stable CM volume at the fovea (Fig. 2B). Visual acuity could not be determined in this patient.

Patient M-B was diagnosed at the age of 14.5 years, with one of the most extended CM of our cohort. An initial 6-month combination therapy of oral acetazolamide with topical brinzolamide was prescribed. The follow-up OCT examination showed a 19% and 23% decrease of the CM surface area on the right and left eyes, respectively (Fig. 2C and Supplementary Table S2). The best-corrected visual acuity remained stable at 20/50 on the left eye and improved from 20/50 to 20/40 on the right eye. This encouraging result led to the prescription of another 6 months of treatment. Unfortunately, the follow-up OCT examination presented with a return to baseline levels of CM surface area (Fig. 2D).

### CM remains relatively stable in teenage and young adult CS patients in the absence of treatment

**Figure 3:**
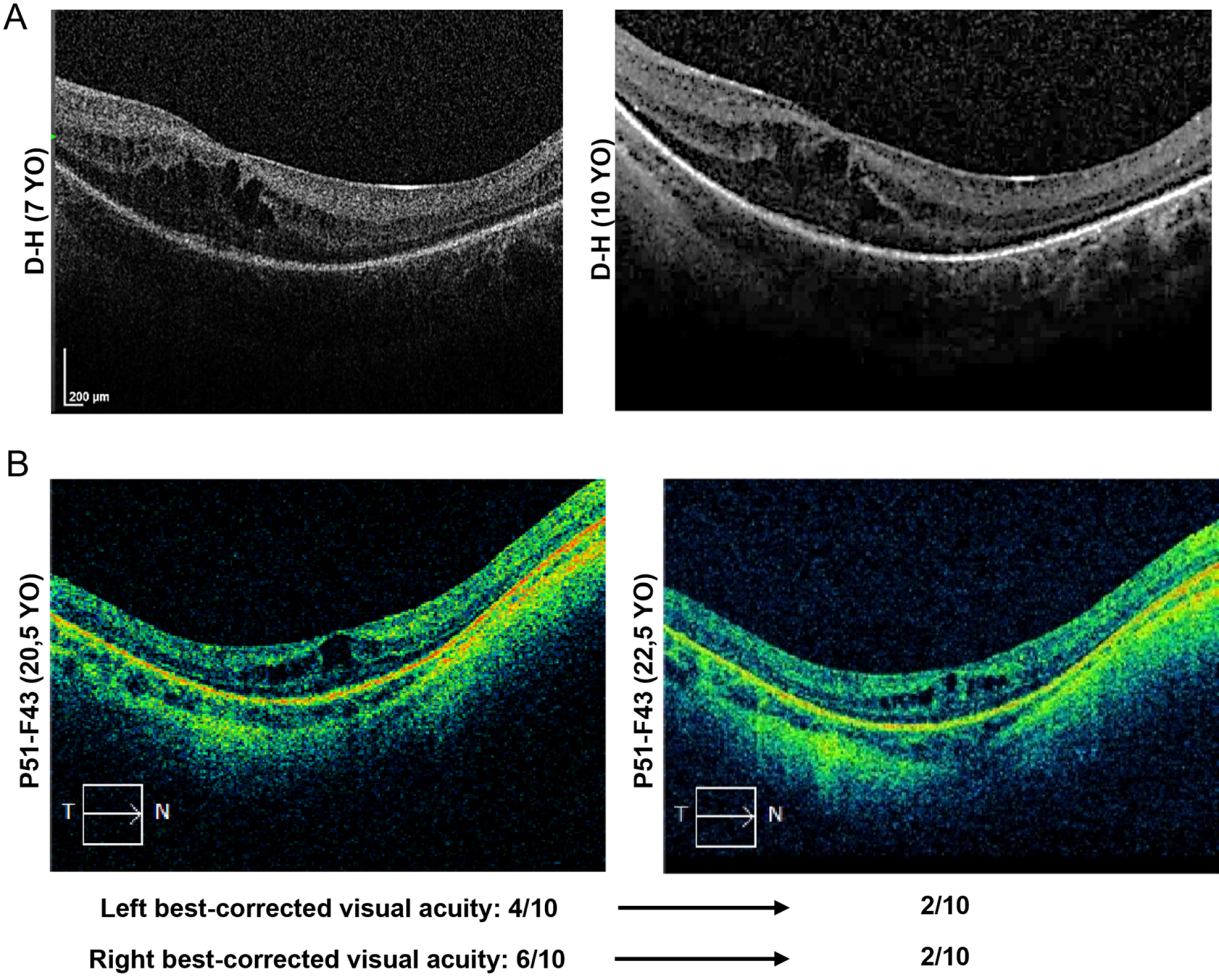
Evolution of CM in absence of treatment. (**A**) In absence of acetazolamide efficacy, patient H–D was left untreated from the age of 6 years until the age of 10 years. Yearly OCT examinations showed little variation in CM manifestations. (**B**) In absence of treatment, patient P51-F43 showed a reduction of CM volume and foveal thickness between the ages of 20.5 and 22.5 years. Conversely, the improvement in CM manifestations was accompanied with a decrease in best-corrected visual acuity.

In two patients, H–D and P51-F43, multiple OCT examinations were performed within a 2- to 4-year period in the absence of treatment, thereby allowing the description of the natural course of CM evolution in CS patients. While patient H–D showed little changes from 7 to 10 years of age (Fig. 3A), patient P51-F43 showed an apparent spontaneous reduction of the CM surface area from the age of 20 to 22 years (Fig. 3B). This reduction was associated with a reduction of the best-corrected visual acuity from 4/10 to 2/10 for the left eye and 6/10 to 2/10 for the right eye.

### The presence and extension of CM correlated with the thickness of photoreceptor-related layers and pigmentary changes

**Figure 4:**
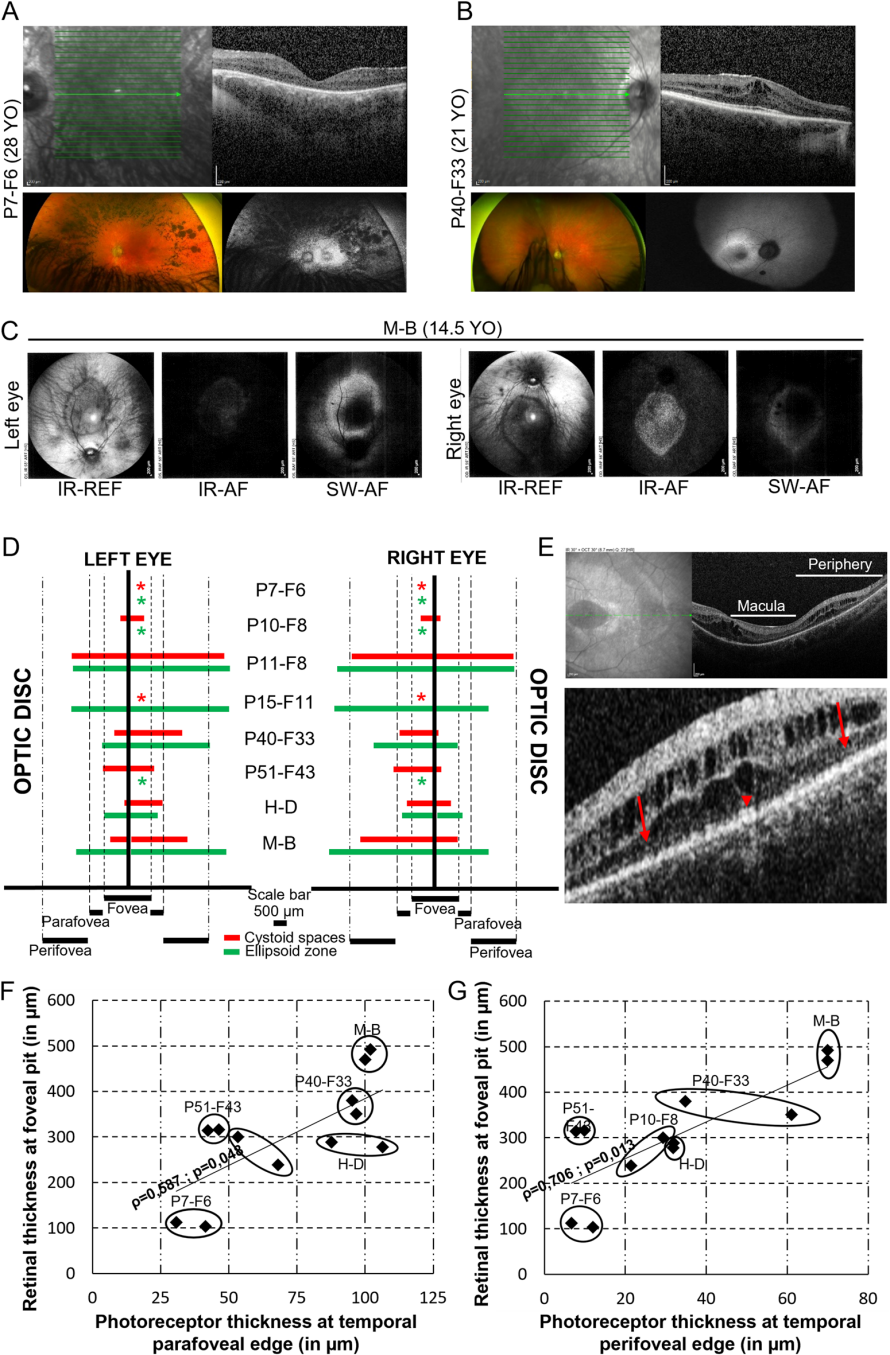
Correlative analyses between natural course of CM and photoreceptor dystrophy in CS patients. (**A**) Patient P7-F6 who presented with no CM had the most reduced ONL in the macula and the most affected retinal periphery in terms of pigment migration. (**B**) In contrast, patient P40-F33 who presented with rather severe CM had no pigment migration on fundus images and a rather preserved ONL thickness. (**C**) Near-infrared (IR-AF) and short-wave autofluorescent fundus (SW-AF) show a hyper-reflective ring and surface, respectively. (**D**) Graphical representation of macular areas affected with cystoid spaces (red) and preserved photoreceptor ellipsoid zone (green). Except for patient P15-F11, wider areas of preserved ellipsoid zone were associated with wider areas of cystoid spaces. Red: TLCS; Green: TLEZ; *: not detected. (**E**) Patients P40-F33 and H–D showed cystoid spaces in the retinal periphery as well as the macula. Lower panel: magnification of the retinal periphery affected with cystoid spaces in the ONL and INL, while showing a relatively preserved ellipsoid zone. Interestingly, surrounding areas where the ellipsoid zone was not preserved did not show cystoid spaces. Local areas of preserved ellipsoid zone in the retinal periphery were not seen in other CS patients. (**F**,**G**) Scatter plots of the photoreceptor thickness at the parafoveal (**F**) or perifoveal (**G**) edge as a function of the retinal thickness at the foveal pit in patients who did not receive treatments and that were examined with the Spectralis system. Both plots show that the more preserved are photoreceptor-related layers, the more elevated is the fovea. Both plots were subjected to Spearman’s statistics and a statistically significant positive correlation was found. ρ: Spearman’s correlation coefficient; p: p-value.

According to our findings, the frequency of CM seems relatively higher in CS patients than RP patients. In this study, we attempted to uncover whether there are specificities to the CS-associated rod-cone dystrophy that could explain the high prevalence of CM in these patients. First, we addressed whether CM and its extension could be related to the degree of pigment migration from the periphery to the fovea observed on color fundus imaging. We found that eyes with a higher CM volume and transversal length of cystoid spaces are associated with fewer pigmentary changes near the macula (Fig. 4A,B and Supplementary Fig. S1). Patient M-B, who showed the most prominent CM, had nearly no pigment migration visible on both eye fundi. Patient H–D who had the widest transversal cystoid length also presented with pigmentary changes very distant from the macula. Among the two most affected patients with pigment migration, one (P10-F8) had mild CM that was only located at the fovea while the other one (P7-F6) was free of CM (Fig. 4A and Supplementary Fig. S1). However, patients P40-F33, who had more extensive CM, did not present pigment migration on fundus images (Fig. 4B). Overall, CS patients with mild pigment remodeling appear to be more affected with CM. In this landscape, patient P15-F11 remained an exception with few pigmentary changes, and yet, no cystoid spaces.

To determine the relation between RPE health and the presence of CM, we assessed short-wave autofluorescence (SW-AF) in patients P7-F6, P10-F8, P40-F33, H–D and M-B as well as near-infrared autofluorescence (NIR-AF) in patient M-B. They all presented with a perifoveal hyper-autofluorescent ring of SW-AF combined (P7-F6, P10-F8, H–D) or not (P40-F33, M-B) with extensive peripheral loss of autofluorescence (Fig. 4A–C). No qualitative differences were noted between patient P7-F6 who did not develop CM and the other patients. In addition, SW-AF observations, as well as the NIR-AF in patient M-B, were typical for RP.

In non-syndromic RP cases, about 79% of cystoid spaces locate to areas of rather well-preserved outer retinas where the EZ can be discriminated^40^. We also found that eyes with the broadest preserved EZ had more extensive cystoid changes (Fig. 4D). In contrast, all three patients for whom the EZ was no longer present had mild (P51-F43 and P10-F8) or no (P7-F6) cystoid spaces. Patient H–D was very peculiar as he presented with a large area of cystoid spaces outside the macula in addition to CM (Fig. 4E). We also performed a multivariate correlation analysis between the OCT parameters that were measured and found a significantly positive correlation between the foveal elevation and (1) the combined thickness of photoreceptor-related layers at the parafoveal and perifoveal regions, and (2) the TLEZ (Supplementary Fig. 2 and Fig. 4F–G). In patient P51-F43, the spontaneous reduction of CM at the age of 22 years was associated with a reduction in the patient’s best-corrected visual acuity and to a progressing atrophy of photoreceptor-related layers on OCT imaging. These results suggest that CS patients may develop CM during the early phase of photoreceptor dystrophy and that CM may resolve has photoreceptor dystrophy progresses.

### Absence of genotype–phenotype correlation in the OCT outcome

It has previously been speculated that the type of *VPS13B* pathogenic variant may be responsible for the diversity and severity of ophthalmic features in CS^26^. Considering the spectrum of changes observed in OCT, we inquired whether a genotype–phenotype correlation could exist. Taking into account the recessive nature of CS and the fact that pathogenic variants included in this correlation are truncating variants, one would expect a gradient of severity along the 3’ to 5’ axis in relation to the functional domains that are encoded^41–43^ (Supplementary Fig. S3A). However, no such correlation was found neither in these nor previously reported patients (Supplementary Table S3 and Supplementary Fig. S3B-D). The correlation was performed within three different age groups: 1–5 (Supplementary Fig. S3B), 6–25 (Supplementary Fig. S3C) and 26–50 years old (Supplementary Fig. S3C). Corroboratively to our previous data suggesting that CM may resolve spontaneously over time, only one out of six patients in the 26- to 50-year-old group presented with CM. However, three of these patients without CM had a severe loss of macular photoreceptors.

## Discussion

CS-associated retinopathy has been extensively described using ophthalmoscopic and electroretinographic examinations^13,17,18,20^. The use of OCT to characterize CS patients' retinal features is very recent and allowed the identification of CM as an early onset feature of CS as well as a better assessment of the severity of the photoreceptor dystrophy^23–25^. In addition to providing information on the disease progression, OCT examination is easier to perform on CS patients than ERG, or even visual acuity tests, due to the limited participation of some of them. Successful OCT examination was even performed in a 1-year-old CS infant^23^. However, OCT imaging documents retinal structures only, and there is no systematic correlation between structural and functional changes in rod-cone dystrophies. While functional analyses through ERG is most often impossible to perform without anesthesia in CS patients^17,27^, visual acuity testing should be attempted even in cases of poor cooperation using simple approaches such as the Rossano-Weiss chart.

The presence of CM in CS is not surprising since CS-associated retinopathy presents with classical features of RP and that CM is a common complication of the condition^38^. In this study, we have found that CM affected eight out of ten CS patients. Including previous case reports, the incidence of CM in CS is of about 80% and is the highest ever reported for a group of rod-cone dystrophy^33^. In patients before 30 years of age, the incidence is even higher and reaches 90%. In RP, the overall incidence of CM is estimated at 30%^32^. Some disease-causing genes are associated with higher incidence, especially *CRB1,* which associates with CM in about 50% of the cases ^44^. Interestingly, some cases with pathogenic variants in *CRB1* have also been associated to a form of CM which evolved into macular atrophy with disappearance of the cystoid spaces in the follow-up OCT examinations^45,46^. To date, no RP genes cause an incidence of CM as high as the incidence related to *VPS13B*. Even an atypical none-CS patient with *VPS13B* disease-causing variants was identified with CM^10^. However, there may be a bias in the report of CS-associated macular changes since all published studies are so far of retrospective nature. The actual frequency of CM in CS may therefore be lower than we estimate. Indeed, only patients with macular changes may be clinically investigated and reported. Nevertheless, the frequency of CM may be higher in CS than other forms of RP for several reasons: (1) it may occur with an earlier onset, and (2), it may be the result of the loss of a specific function of VPS13B related to retinal cell adhesion as it occurs in X-linked retinoschisis^47^. This second hypothesis is supported by the absence of leakage during fluorescein angiography in two CS patients^24,27^. While additional angiographic data are required to conclude on the etiology of CM in CS-associated retinopathy, it has been hypothesized that macular cysts in CS may be due to abnormalities in the retinal architecture rather than a breakdown of the blood-retinal barrier and the intraretinal accumulation of fluids^30^. In addition, CS patients sometimes have severe myopia with posterior staphyloma, a condition that has been associated with retinoschisis and may therefore contribute to CM in CS-associated retinopathy. However, in our case series, non-staphylomatous eyes also presented with CM.

Of clinical interest, CM seems less likely to be identified in older patients with more advanced retinal dystrophy. We previously reported the cataract surgery of two CS siblings in their 40 s with a best-corrected visual acuity between 1/5 and 1/10 (Rossano-Weiss) who had no CM before or after surgery^21^. In this study, we found that the presence and severity of cystoid spaces in the retina seemed to be negatively correlated with photoreceptor dystrophy progression. Indeed, the volume of cystoid spaces was positively correlated to the preservation of the EZ. In the absence of treatment, a patient showed a decrease in the volume of CM over 2 years and this decrease was associated with a reduction of the EZ, a reduction of the ONL in the macula and a decrease in visual acuity. In the case of patient P7-F6, who had the most reduced photoreceptor-related layers in the macula and lowest visual acuity at 28 years of age, it is likely that CM had already resolved at the time of the first OCT examination. The observations made in our study are in line with the fact that CM is more commonly seen in early stages rather than in late stages of RP with extensive rod-cone degeneration^48^.

While CM may resolve spontaneously within one or several decades without therapeutic intervention, improving young CS patients' central vision remains of significant interest since most of them already suffer from severe peripheral dystrophy. In the absence of data, CM may cause decreased central VA in CS patients and we therefore aimed at reducing CM volumes using CAIs. Similarly to a previous case report^24^, the use of a systemic CAI did not lead to functional or anatomical improvement of CM in two CS children. A combination of systemic and topical CAI also failed to do so in the long term in another patient, even though a transient improvement was detected after 6 months of treatment. In patient H–D, CM remained relatively stable from the age of 7 to 10 years without treatment. Our work emphasizes previous data (Supplementary Table S3) suggesting that CAIs may have a limited effect in reducing and stabilizing cystoid volumes in CS patients^24,25,30^. Other therapeutic strategies have been assessed but should be considered with caution as some still remain controversial^38^. In addition, the etiology of CM in CS may differ from that of CME and treatments found effective in RP patients may not be in CS patients.

Among all CS patients examined with OCT imaging, patient P15-F11, despite being at an age where patients are usually severely affected, was from far the one with milder retinal phenotypes both in terms of photoreceptor dystrophy and CM. Understanding the etiologic factors that contribute to his milder phenotype could help in the management of CS-associated retinopathy, especially when considering that there is no genotype–phenotype correlation. CS being a multisystemic disorders, deciphering physiological and environmental factors that come into play may require a very complex study design and the recruitment of a larger patient cohort. Two physiological aspects have so far been extensively investigated in our cohort: neutropenia^11^ and metabolic aspects^12^. Neutropenia is transitory in CS patients and correlation to ophthalmic findings is therefore not possible. When looking at previously collected metabolic data^12^, we found that patients with more severe metabolic changes, also tend to develop more severe retinal changes (Supplementary Table S4). Patient P7-F6, who had the thinnest photoreceptor-related layers in the macula and the lowest best-corrected visual acuity, was as well the only patient to be diagnosed with hepatic steatosis and the one with the highest blood pressure. He also had one of the lowest HDL levels but normal glycemia at 120 min during an oral glucose tolerance test (GTT). Patient P51-F43 who showed complete loss of EZ and progressive decrease of CM, macular photoreceptors, and best-corrected visual acuity, had abnormally elevated glycemia as well as low HDL and high triglyceride levels. Interestingly, patient P15-F11 with no macular changes was the one with the lowest glycemia at 120 min during oral GTT. Metabolic data were not systematically collected in our cohort due to low patient compliance with the tests and more data are required to evaluate a potential correlation between metabolic and retinopathic aspects in CS.

Several limitations should be acknowledged in this study. Although being large in regard to ultra-rare diseases such as CS, our cohort remains small (n = 10), and more OCT examinations are required to draw definite conclusions. Our study and other data on CS-associated macular changes are either of retrospective nature or case reports. The initial or early stages of the condition are often missing, and correlation between OCT imaging and fluorescein angiography has rarely been performed to determine the etiology of the cystoid spaces. There is still no guaranty that all CS patients with CM do not present with intraretinal leakage. To help manage CS-associated CM, a prospective longitudinal study with yearly OCT and angiography examinations including CS patients in their first or second year of age would be of great benefit.

Overall, our study suggests that CM is a frequent finding in CS with a prevalence of about 80%. The severity of CM, but not necessarily its presence (P15-F11), may be positively correlated to the presence of macular and peripheral photoreceptors, and CM may resolve spontaneously during adulthood as photoreceptor dystrophy progresses towards the fovea. Importantly, CAI treatments may not be sufficient to improve the condition in CS patients. A significant drawback to the development of therapeutic approaches to alleviate the CS-associated retinal pathology is the lack of knowledge on VPS13B functions in the retina and models to assess therapeutic strategies. We recently reported the creation of a mouse model for Cohen syndrome^6^. A hypermature form of cataract strongly affected the retina of this model through intra ocular inflammation in a manner that does not embody CS-associated retinopathy. To study CS-associated retinopathy, a non-cataractous subline of this model was then isolated through selective breeding^21^. The ophthalmic characterization of this model should provide valuable information on the retinal cell types with critical VPS13B functions, an initial understanding of these functions, and, hopefully, a faithful model to assess therapeutic strategies.

## Supplementary Information

Supplementary Information 1.

Supplementary Information 2.

Supplementary Information 3.

Supplementary Information 4.

Supplementary Information 5.
