# Supplementary material for: Cystoid maculopathy is a frequent feature of Cohen syndrome-associated retinopathy
Source: Sci Rep. 2021 Aug 12;11:16412. doi: 10.1038/s41598-021-95743-8 (PMC8361024; doi:10.1038/s41598-021-95743-8)
Supplement: Supplementary file 1 — Supplementary Information 1. [file 41598_2021_95743_MOESM1_ESM.pdf]

Supplementary Information

Cystoid maculopathy is a frequent feature of Cohen syndrome-associated retinopathy

Pierre-Henry Gabrielle[1•], Laurence Faivre[2,3,4•], Isabelle Audo[5,6•], Xavier Zanlonghi[7], Hélène Dollfus[8,9], Alberta A. H. J. Thiadens[10], Christina Zeitz[5], Grazia M. S. Mancini[11], Yaumara Perdomo[8,9], Saddek Mohand-Saïd[5,6], Eléonore Lizé[2], Vincent Lhussiez[2], Emeline F. Nandrot[5], Niyazi Acar[12], Catherine Creuzot-Garcher[1,12], José-Alain Sahel[5,6,13], Muhammad Ansar[14], Christel Thauvin-Robinet[2,3,15], Laurence Duplomb[2,3], Romain Da Costa[2,3*]

Corresponding author

Romain Da Costa Ph.D.,

Equipe GAD, Inserm UMR1231, Bâtiment B3,

15 boulevard du Maréchal de Lattre de Tassigny

21079 Dijon, Cedex, France

Tel: +33 3 80 39 32 38

Fax: +33 3 80 29 32 66

e-mail: romain.dacosta@chu-dijon.fr

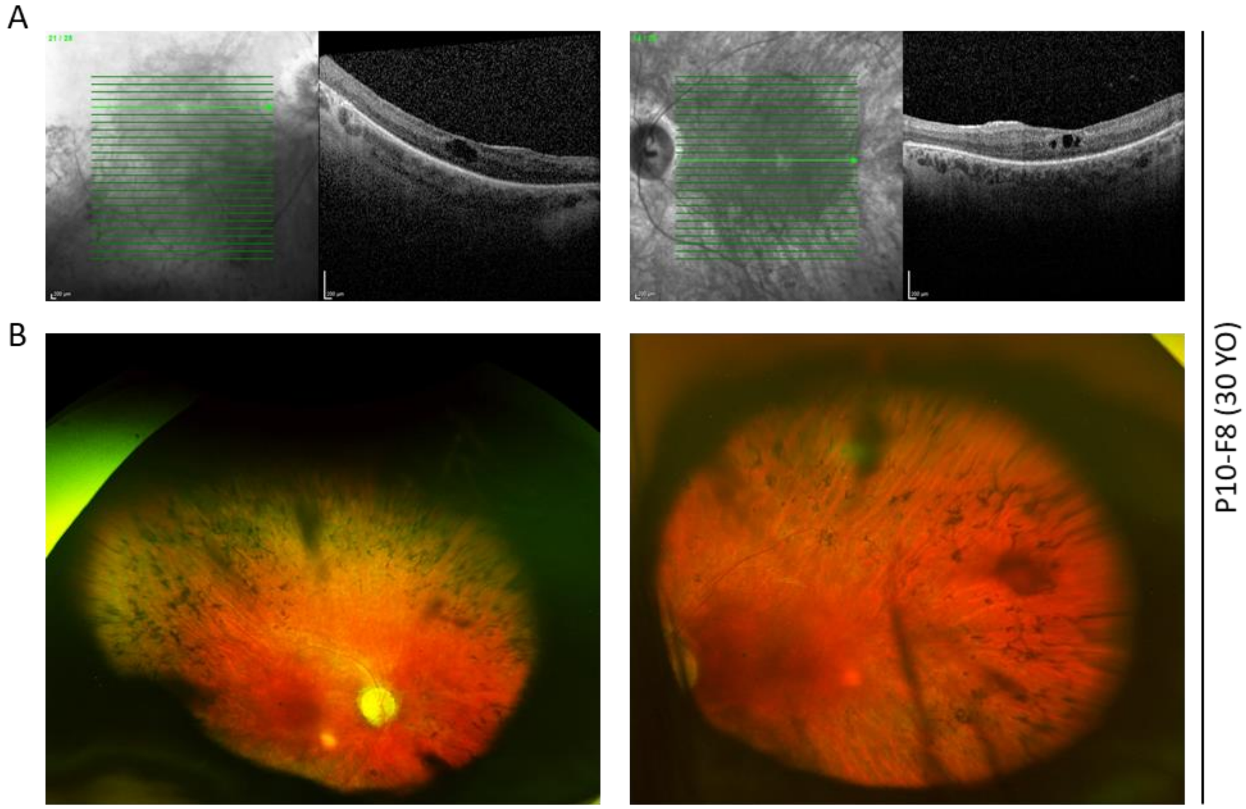

Supplementary Figure S1. CM in relation to fundus pigment migration in patient P10-F8. (A) Transfoveal OCT images from patient P10-F8 at the age of 30 years. **(B)** Fundus images showing peripheral pigment deposits at the same age.

A

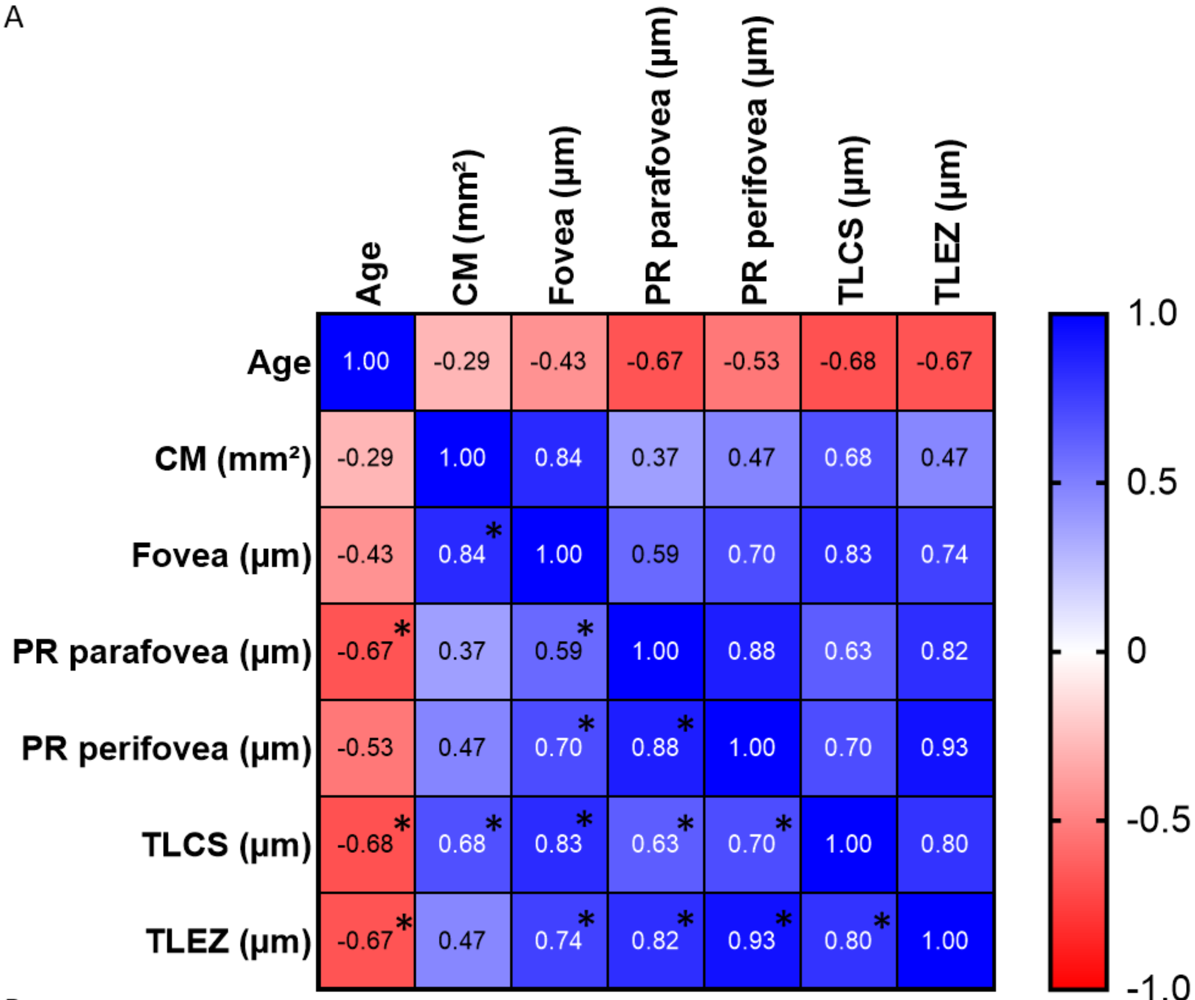

B

**P values**	Age	CM (mm²)	Fovea (µm)	PR parafovea (µm)	PR perifovea (µm)	TLCS (µm)	TLEZ (µm)
Age							
CM (mm²)	0.3627						
Fovea (µm)	0.1656	**0.0012**					
PR parafovea (µm)	**0.0194**	0.2290	**0.0489**				
PR perifovea (µm)	0.0780	0.1202	**0.0135**	**0.0003**			
TLCS (µm)	**0.0173**	**0.0170**	**0.0014**	**0.0304**	**0.0145**		
TLEZ (µm)	**0.0197**	0.1264	**0.0083**	**0.0022**	**0.0001**	**0.0035**	

Supplementary Figure S2. Multivariate non-parametric Spearman's correlation analysis of the measured OCT parameters. (A) Correlation matrix displaying Spearman's correlation coefficients. *: $p<0.05$. **(B)** Table of the exact p-values calculated for each coefficient.

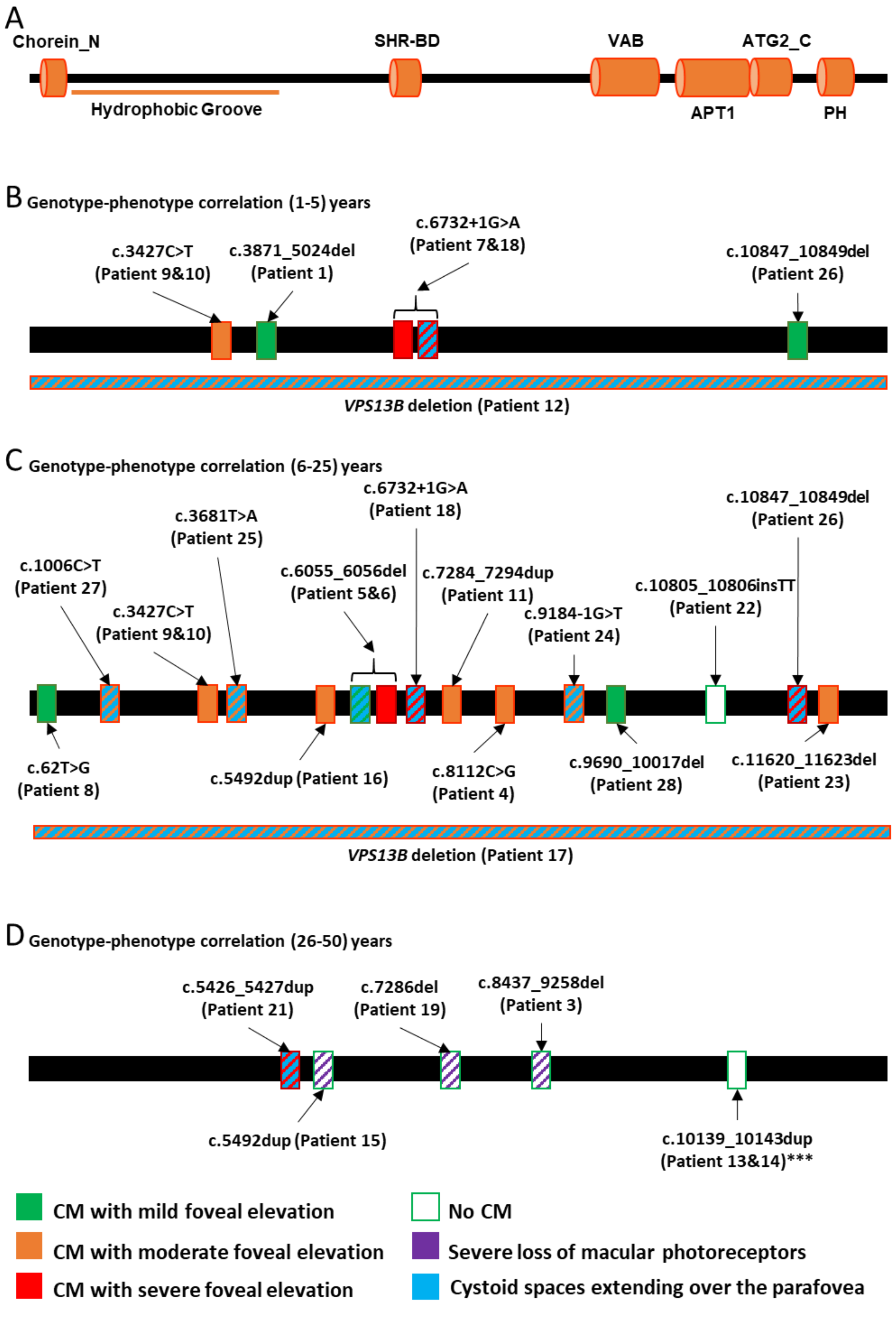
A
Chorein_N
SHR-BD
VAB
ATG2_C
Hydrophobic Groove
APT1
PH
B Genotype-phenotype correlation (1-5) years
c.3427C>T (Patient 9&10)
c.3871_5024del (Patient 1)
c.6732+1G>A (Patient 7&18)
c.10847_10849del (Patient 26)
VPS13B deletion (Patient 12)
C Genotype-phenotype correlation (6-25) years
c.1006C>T (Patient 27)
c.3681T>A (Patient 25)
c.3427C>T (Patient 9&10)
c.6055_6056del (Patient 5&6)
c.6732+1G>A (Patient 18)
c.7284_7294dup (Patient 11)
c.9184-1G>T (Patient 24)
c.10805_10806insTT (Patient 22)
c.10847_10849del (Patient 26)
c.62T>G (Patient 8)
c.5492dup (Patient 16)
c.8112C>G (Patient 4)
c.9690_10017del (Patient 28)
c.11620_11623del (Patient 23)
VPS13B deletion (Patient 17)
D Genotype-phenotype correlation (26-50) years
c.5426_5427dup (Patient 21)
c.7286del (Patient 19)
c.8437_9258del (Patient 3)
c.5492dup (Patient 15)
c.10139_10143dup (Patient 13&14)***
CM with mild foveal elevation
CM with moderate foveal elevation
CM with severe foveal elevation
No CM
Severe loss of macular photoreceptors
Cystoid spaces extending over the parafovea

Supplementary Figure S3. Schematic drawing displaying the severity of macular changes of CS patients in association with the disease-causing variants and age. (A) Predicted VPS13B functional domains[41–43]. Chorein_N: N-terminal chorein domain (Pfam 12624) ; SHR_BD: SHR binding domain of vacuolar protein sorting-associated protein 13 (Pfam 06650) ; VAB: Vps13 Adaptor Binding/WD40 domain ; APT1: APT1 domain ; ATG2_C: Autophagy-related protein 2 C-terminal domain; PH: Pleckstrin homology domain. **(B)** Genotype-phenotype correlation from OCT observations in patients from 1 to 5 years of age. **(C)** Genotype-phenotype correlation from OCT observations in patients from 6 to 25 years of age. **(D)** Genotype-phenotype correlation from OCT observations in patients from 26 to 50 years of age. In case of compound heterozygosity, we correlated the 3' most variant with the severity of CM due to the recessive nature of CS. All variants presented here are truncating variants, except for patient H-D who has a homozygous phenylalanine deletion. No gradient of severity was identified along the 5' to 3' axis of *VPS13B*.
